# Laboratory Investigation on the Impact Force of Large Boulders in Debris Flows

**DOI:** 10.3390/s26061983

**Published:** 2026-03-22

**Authors:** Wei Yi, Bin Yu, Qinghua Liu, Jianchun Hu, Jun Zhou

**Affiliations:** 1 School of Tourism and Urban-Rural Planning, Xichang University, Xichang 615000, China; 2 State Key Laboratory of Geological Disaster Prevention and Geological Environment Protection, Chengdu University of Technology, Chengdu 610059, China; 3 School of Ecology and Environment, Chengdu University of Technology, Chengdu 610059, China; 4 The Eighth Geological Brigade of Sichuan Province, Xichang 615000, China

**Keywords:** debris flow, large boulder, impact force, dimensional analysis, experimental study

## Abstract

The impact of large boulders transported by debris flows is a primary cause of structural damage to mitigation works. However, quantitative modeling remains difficult because of the scarcity of field measurements and the complexity of the flow medium. In this study, a theoretical model for boulder impact force in debris flows is developed using dimensional analysis based on the Buckingham theorem, subsequently simplified to two dimensionless parameters, and then validated through a series of controlled laboratory experiments. Marble spheres were used as impactors and were released to strike a rigid steel plate under three types of media: clear water, bentonite slurry, and debris flows containing particles of different size classes. The experiments were designed to isolate and quantify the influence of the flow rheology and the suspended solid phase on impact forces. The results show that the impact coefficient c is strongly governed by the debris flow yield stress, bulk density, and the size of suspended particles, following the relationship *c* = 0.183[*τ*/(*rgd*_1_)]^−0.1^(*d*/*d*_0_)^0.05^. Based on this relationship, a generalized formula for calculating boulder impact forces in debris flows is proposed. The model is further evaluated using field monitoring data from Jiangjiagou, Yunnan Province. The back-calculated boulder diameters fall predominantly within the range of 0.1–0.3 m (76.3–86.8%), which is consistent with field observations. These results indicate that the proposed model captures the essential physical mechanisms governing boulder impacts and provides a rational basis for selecting design parameters in debris flow mitigation engineering. The array-type piezoelectric impact sensing system designed in this study achieves high-precision and high-stability measurement of the impact force of large boulders in debris flows, providing a new sensing technology for debris flow impact monitoring.

## 1. Introduction

Debris flows are a common and highly destructive natural hazard in mountainous regions, characterized by long travel distances and high impact energy. They pose a serious threat to the safety and sustainability of infrastructure such as towns and transportation networks [1,2,3,4,5]. The immense kinetic energy carried by debris flows often leads to the destruction or burial of bridges, roads, and other engineering structures along their paths, resulting in significant loss of life and property [6]. Accurate quantification of debris flow impact forces, particularly those exerted by large boulders, is therefore essential for precise hazard assessment and the design of effective mitigation measures [7,8].

The total impact force of a debris flow primarily consists of the dynamic pressure of the flow matrix and the collision force of large boulders [9], with boulder impacts being the dominant factor causing structural failure [10]. However, the sudden occurrence of debris flows and the high-risk nature of field observations make it extremely difficult to obtain high-quality in situ impact force measurements. Existing field datasets remain limited [11] and often exhibit significant scale differences and regional variability. As a result, empirical formulas derived from these data typically lack general applicability and can produce substantial errors when applied to other regions [12].

To overcome the limitations of field studies, physical model experiments have become an important approach for investigating the impact mechanisms of debris flows [13,14,15]. Many researchers have employed flume experiments to study the effects of debris flows on various barriers, such as vertical walls and check dams [16,17,18]. However, these studies often focus on the overall dynamic pressure of the flow matrix or consider only the peak impact force of large boulders. Moreover, key similarity criteria in laboratory experiments, such as the Froude number, differ significantly from those of field prototypes, limiting the direct applicability of small-scale models to real-world scenarios [19,20]. Existing methods for calculating boulder impact forces are mostly based on the momentum theorem or energy conservation principles. Their core parameters, such as impact duration and displacement, are difficult to determine accurately in the complex and heterogeneous debris flow medium, restricting their effectiveness for engineering applications.

Yu et al. [21] successfully developed a dimensional analysis-based model to predict the impact force of rolling stones in air and demonstrated its applicability across different scales. This work provides a critical theoretical foundation for the present study. Building on this approach, the core scientific hypothesis of this research is that the influence of debris flow media on boulder impact force can be represented by introducing a correction coefficient into a basic impact model. This coefficient primarily depends on the bulk density, yield stress, and particle size distribution of the debris flow.

Accordingly, the objectives of this study are (1) to derive a theoretical model for boulder impact force incorporating debris flow medium parameters using dimensional analysis; (2) to design and conduct systematic laboratory experiments with water, slurry, and debris flows of varying particle gradations to determine the key parameters of the model; and (3) to validate the resulting formula against field monitoring data and evaluate its applicability in engineering practice. This study also develops a dedicated sensing system for debris flow impact monitoring, offering practical insights for advancing geohazard monitoring technologies, while providing a more reliable theoretical tool to underpin the sustainable and resilient design of debris flow mitigation structures.

## 2. Model Derivation

The impact of a moving block is essentially a process of work done by the impact force. The change in total kinetic energy is equal to the product of the impact force and the corresponding impact displacement [21]:

(1) $$F=\frac{△E_{k}}{l}$$

where (F) is the impact force (N), E_k_ is the total kinetic energy (J), and (l) is the impact displacement (m).

In debris flows, large boulders commonly roll during downslope motion. At the instant of impact, the total kinetic energy therefore comprises both translational and rotational components [22,23]. JRA (1983) [24] reported that rotational kinetic energy (Er) is proportional to translational kinetic energy (Et), allowing Er to be estimated from Et.

Let the ratio β = Er/Et. The total kinetic energy can then be expressed as

*E*_*k*_ = (1 + *β*) *Et* (2)

where E_k_ is the total kinetic energy of the impacting block (J), Et is the translational kinetic energy (J), and Er is the rotational kinetic energy (J). According to JRA [24], this ratio does not exceed 10% in most field conditions. However, flume experiments by Chau et al. [23] showed that when the slope angle reaches 40°, β may increase to 0.4. This value can be regarded as an upper bound for the rotational energy fraction of a spherical impactor.

The translational and rotational kinetic energies are defined as

*Et* = 0.5*MV*^2^ (3)

*Er* = 0.5*Iω*^2^ (4)

where M is the mass of the impacting body kg, V is its translational velocity m/s, I is the moment of inertia (kg·m^2^), and ω is the angular velocity (rad/s). For a spherical block, the moment of inertia is given by I = 0.4 Mr^2^, where r is the radius (m). The rotational kinetic energy can therefore be related to the translational velocity through the block geometry.

The impact displacement arises from the interaction between the impacting body and the target structure. It is governed by the mechanical properties of both materials, in particular their elastic moduli and Poisson’s ratios. According to the Hertzian theory of elastic contact, the combined effect of the elastic modulus and Poisson’s ratio of the two contacting bodies can be represented by an equivalent elastic parameter (E). This parameter characterizes the contact stiffness and controls the magnitude of the resulting impact force:

(5) $$E=\frac{E_{1}E_{2}}{(1\;-\;u_{2}^{2})E_{1}+{\;E}_{2}(1\;-\;u_{1}^{2})}$$

where *E*_1_ and *E*_2_ are the elastic moduli, and u_1_ and u_2_ are the Poisson’s ratios of the impacting and impacted bodies, respectively. For common engineering materials, Poisson’s ratio typically ranges from 0 to 0.3, giving (1−u_1_^2^) and (1−u_2_^2^) values between 0.91 and 1.0. Following a simplification principle, Equation (1) can be approximated as

(6) $$E=\frac{E_{1}E_{2}}{E_{1}+kE_{2}}$$

Here, k is a coefficient that reflects the relative contribution of the elastic moduli of the impacting body and the target body to the equivalent elastic parameter E. It accounts for the stiffness contrast between the two contacting materials.

To establish a dimensionless formulation, the key dimensional variables F, E_k_, and E are selected. In the MLT base system, their dimensions are expressed as

{*F*} = {*MLT* − 2}, {*Ek*} = {*ML*2*T* − 2}, {*E*} = {*ML* − 1*T* − 2} (7)

According to the Buckingham π theorem, the number of independent dimensionless groups equals the number of physical variables minus the number of fundamental dimensions. By incorporating the geometric parameter sin α, the governing dimensionless group can be formulated as

(8) $$\Pi_{1}=\frac{F}{{\left(EE_{k}^{2}\right)}^{1/3}}$$

As Π_1_ is functionally related to sin α, that is, Π_1_ = f(sin α), the impact problem can be recast in dimensionless form as

*F* = *cE*^1/3^*E*_k_^2/3^*f*(sin *a*) (9)

where c is a functional coefficient.

Combining Equations (3)–(9), the impact force can be rewritten as follows:

*F* = *c*(1 + *β*)^2/3^*E*^1/3^*M*^2/3^*V*^4/3^*f*(sin *a*) (10)

Yu et al. [21] conducted experiments on rolling stones in air without considering rotational kinetic energy and found that *E* = *E*_1_*E*_2_/(*E*_1_ + 2*E*_2_). They also observed that the impact force F in air correlates well with [*E*_1_*E*_2_/(*E*_1_ + 2*E*_2_)]^1/3^, *M*^2/3^, *V*^4/3^, and (sin *a*)^1/2^, giving the empirical expression:

*F* = 0.35 [*E*_1_*E*_2_/(*E*_1_ + 2*E*_2_)]^1/3^*M*^2/3^*V*^4/3^(sin *a*)^1/2^ (11)

Assuming that the functional relationship between boulder impact force and [*E*_1_*E*_2_/(*E*_1_ + *2E*_2_)]^1/3^, *M*^2/3^, *V*^4/3^, and (sin *a*)^1/2^ in debris flow media is similar to that in air, a dimensionless model considering both rotational and translational kinetic energy for boulders in debris flows is proposed:

*F* = *c*(1 + *β*)^2/3^[*E*_1_*E*_2_/(*E*_1_ + 2*E*_2_)]^1/3^*M*^2/3^*V*^4/3^(sin *a*)^1/2^ (12)

In this study, laboratory experiments were conducted to determine the values of the model parameters c and β for debris flow boulder impacts.

## 3. Experimental Design

### 3.1. Experimental Setup

**Figure 1:**
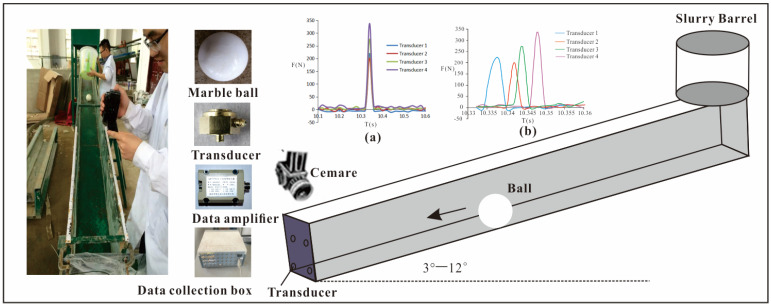
Experimental apparatus.

The experimental system is illustrated in Figure 1. Its core component is a linear flume with dimensions of 10 m × 0.38 m × 0.5 m. To precisely control the impact location, a guiding channel measuring 8 m × 0.2 m × 0.2 m is installed along the center of the main flume, ensuring that the boulder consistently strikes the central region of the target plate.

### 3.2. Design and Performance of the Sensing and Measurement System

Specially optimized for the transient and high-noise characteristics of debris flow multiphase impacts, the self-developed array-type piezoelectric impact force measurement system in this study offers a novel sensing tool for laboratory investigations of debris flow impact mechanics and technical support for real-time field monitoring of debris flow hazards. Its core innovations lie in three key aspects: sensor array configuration, wide-range design, and systematic signal processing. Constructed around QSY8301-01 piezoelectric force sensors, the system integrates a four-channel data acquisition unit and a dedicated signal conditioning module, enabling accurate capture of transient impact forces exerted by large boulders in debris flows.

The system was designed to achieve high measurement accuracy and rapid dynamic response, which are essential for short-duration impact events. Comprehensive characterization was carried out, including static calibration, dynamic performance assessment, frequency response analysis, signal processing evaluation, and measurement uncertainty quantification.

All key parameters and performance indicators of the measurement system were experimentally calibrated. This ensures the reliability and accuracy of the recorded impact force data. The main specifications are summarized in Table 1.

#### 3.2.1. Sensor Selection and Assembly of the Measurement System

The impact force measurement system consists of QSY8301-01 piezoelectric force sensors, a rigid load-transfer plate, a four-channel high-speed data acquisition unit, a signal conditioning module, and a host computer. When the impacting block strikes the impact plate, the transient force is transmitted through the rigid plate to the piezoelectric sensors. The mechanical signal is converted into an electrical charge signal, which is then transformed into a voltage signal by the conditioning module. The voltage output is synchronously recorded by the data acquisition system and transferred to the host computer for post-processing, from which the impact force time history and peak force are obtained.

Four piezoelectric sensors were installed between the impact plate and the rigid backing plate in a rectangular array. Two measurement ranges, 50–500 N and 500–5000 N, were combined to achieve an overall effective range of 200–20,000 N. This configuration accommodates impacts associated with different kinetic energy levels. The data acquisition frequency was set to 5 kHz, corresponding to a sampling interval of 0.0002 s. This ensures temporal synchronization among the four channels and eliminates timing discrepancies in peak force identification (Figure 1a,b).

The initial force fluctuation and associated measurement uncertainty were analyzed. For a single sensor, the baseline fluctuation ranges from −20 N to +20 N. In theory, the combined extreme range for four sensors is −80 N to +80 N. Experimental data show that the initial force is typically confined within ±20 N and does not exceed ±40 N under extreme conditions. The maximum recorded impact force in this study was 15,583 N. When the peak impact force is below 800 N, the relative error induced by the baseline fluctuation may reach 5–18%. For peak forces above 800 N, the measurement error remains within 5%. This multi-channel array-type sensing configuration can be extended to impact force measurement of other geohazards such as rockfalls and landslides, providing a design reference for the engineering application of geohazard monitoring sensors.

#### 3.2.2. Static Calibration and Determination of the Calibration Curve

**Figure 2:**
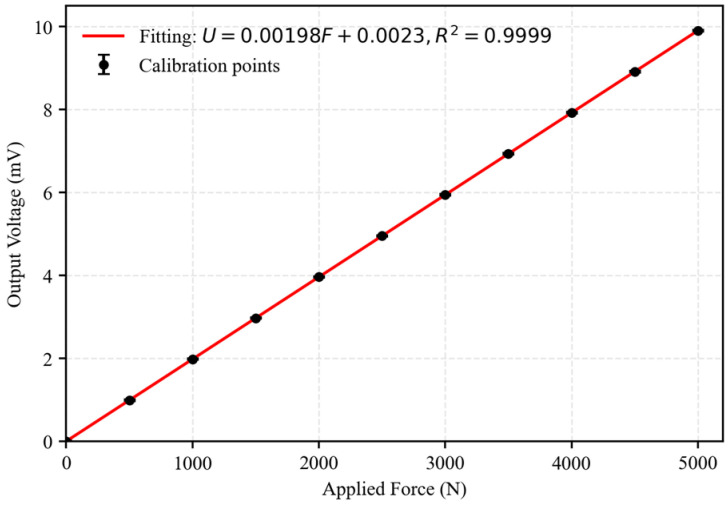
Static calibration curve of the QSY8301–01 piezoelectric force sensor.

To eliminate manufacturing tolerances, installation eccentricity, and signal transmission errors, all sensors were subjected to full-range static calibration. A standard force calibration rig was used to apply stepwise loads from 0 to 5000 N, with increments of 500 N, resulting in 11 calibration points. Each load level was maintained for 30 s to ensure stability. The output voltage was recorded at each level, and every point was measured three times to obtain an average value and reduce random error. As shown in Figure 2, the sensor output voltage exhibits a strictly linear relationship with the applied force. Linear regression yields the static calibration equation:

*U* = 0.00198*F* + 0.0023 (13)

where U is the sensor output voltage (mV) and F is the applied force (N). The coefficient of determination is R^2^ = 0.9999, indicating excellent linearity. The linearity error is 0.12%, the hysteresis error is 0.08%, and the repeatability error is ±0.02 mV. All values are superior to the manufacturer’s specification (±0.5% FS). These results confirm a stable and linear response over the full measurement range, providing a reliable basis for dynamic impact force measurements.

#### 3.2.3. Dynamic Performance and Frequency Response Analysis

**Figure 3:**
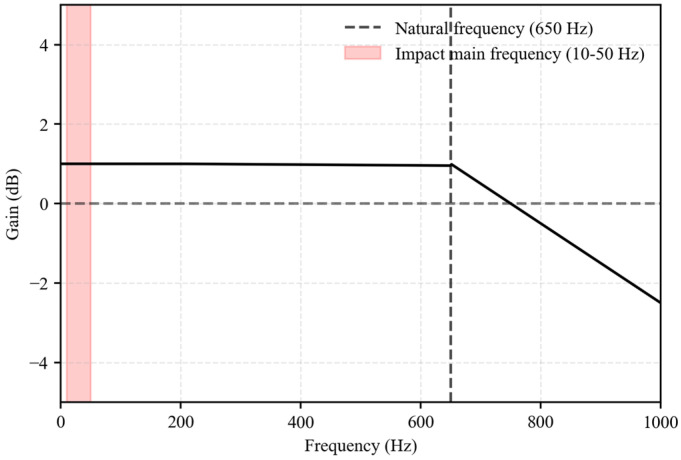
Frequency response curve of the QSY8301–01 force sensor.

The impact of large debris flow boulders is a transient process occurring on the millisecond scale. Spectral analysis based on high-speed camera recordings shows that the dominant frequency content of the impact signal lies between 10 and 50 Hz (This spectral analysis data is obtained from the piezoelectric force sensor system with a sampling frequency of 5 kHz). The sensors must therefore provide a rapid dynamic response and a sufficiently wide frequency bandwidth. Dynamic performance was evaluated through frequency response testing and step-response analysis. An impedance analyzer was used to conduct a frequency sweep from 0 to 1000 Hz, and the results are shown in Figure 3. The natural frequency of the sensor is 650 Hz. Within the range of 0–200 Hz, the amplitude–frequency response remains flat, with a gain fluctuation of less than 0.5 dB. The phase–frequency response is linear, indicating negligible phase distortion. The dominant impact frequencies (10–50 Hz) lie well within the effective bandwidth and are far below the natural frequency. Resonance-induced distortion is therefore avoided, ensuring accurate acquisition of transient signals.

**Figure 4:**
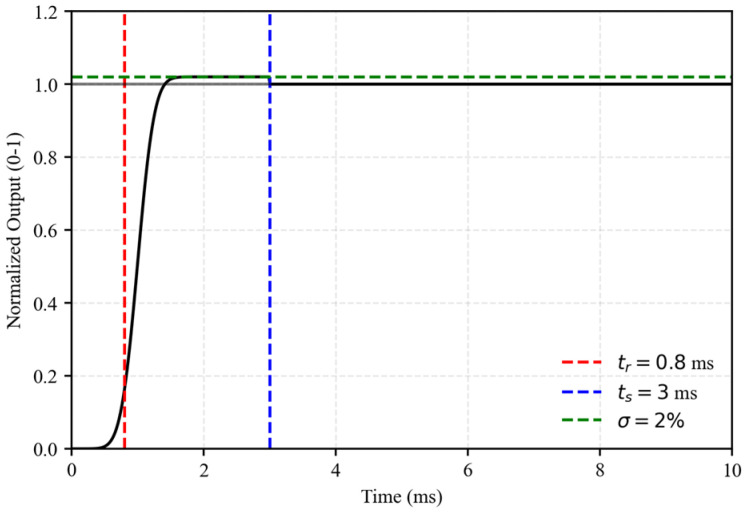
Dynamic step response curve of the QSY8301–01 force sensor.

A drop-weight device was used to generate an ideal half-sine step force to further assess the dynamic response. The results are presented in Figure 4. The rise time Tr is 0.8 ms, defined as the time required to increase from 0.1 to 0.9 of the steady-state value. The overshoot σ is less than 2%, and the settling time Ts is 3 ms. These indicators demonstrate that the sensor can rapidly capture millisecond-scale impact peaks without overshoot, fully satisfying the dynamic measurement requirements for debris flow boulder impacts.

#### 3.2.4. Impact Signal Processing and Validation

**Figure 5:**
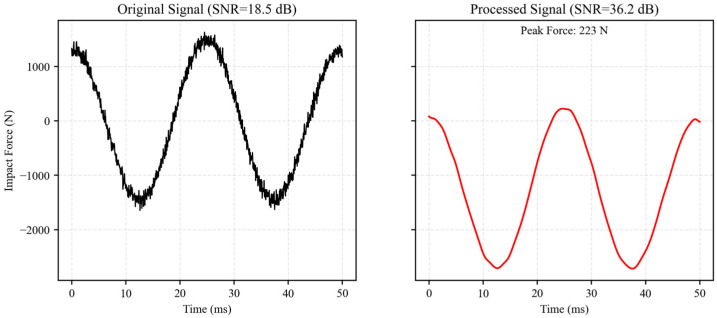
Comparison of debris flow impact signals before and after processing.

During laboratory measurements, the recorded impact signals inevitably contained high-frequency noise. This noise mainly originated from pump vibration, particle friction, and electronic interference. A structured signal processing procedure was therefore implemented, including baseline correction, low-pass filtering, and multi-channel data fusion. The effectiveness of the processing is illustrated in Figure 5. The raw signal exhibits pronounced high-frequency spikes, with a signal-to-noise ratio (SNR) of 18.5 dB, which obscures peak force identification. After processing, the spikes are fully removed, and the time–history curve becomes smooth. The SNR increases to 36.2 dB, and the peak force identification accuracy improves by 4.2%. The processed signal also shows clear agreement with the expected physical characteristics of the impact process, confirming the robustness of the adopted procedure.

### 3.3. High-Speed Imaging and Velocity Determination

Exclusively used for measuring the impact velocity of large debris flow boulders (and not involved in the frequency or spectral analysis of impact signals, which are captured by the piezoelectric force sensor system), the 25 fps frame-rate imaging system was employed to record the impact process. It features a temporal resolution of 0.04 s, and spatial calibration was performed using pre-installed equally spaced markers within the flume. Pixel coordinates were converted to physical distances, with the spatial calibration error constrained to ±0.005 m.

The impact velocity was determined from the last two clear frames immediately prior to contact. The critical frame was defined as the final image in which the sphere had not yet touched the target plate. This avoids displacement errors caused by deformation during contact. The instantaneous velocity was calculated using V = ΔS/Δt, where ΔS is the displacement of the sphere center between two consecutive frames and Δt = 0.04 s. Uncertainty analysis shows that the main sources of error are sphere position identification within a frame (±1 pixel) and spatial calibration. The combined uncertainty in velocity measurement is ±0.08 m/s. Given the measured impact velocity range of 1.5–4.2 m/s, this corresponds to a relative uncertainty of 1.9–5.3%. The 25 fps temporal resolution is therefore sufficient for the present experiments. The 25 fps frame-rate imaging data provide accurate impact velocity inputs for the analytical model. They also confirm that the spheres strike the target plate along the flume axis, eliminating interference from oblique impacts. In addition, the footage offers visual evidence for impact angle and rotational motion, providing an independent check on the reliability of the experimental data.

### 3.4. Water-Only Tests

For the water-only experiments, a 15 L plastic container was used as the water supply. Marble spheres with a mass of 1.56 kg served as the impacting bodies, while a steel plate was employed as the target. To protect the force sensors from water damage, they were multilayer-sealed using impermeable plastic film. After assembling the apparatus, each marble sphere was placed stationary on the track above the flume. Water was then poured from the container above the sphere, causing it to roll and impact the steel plate under submerged conditions (Figure 1). The flow velocity in the experimental flume was determined using a combination of the weir discharge equation and 25 fps frame-rate imaging analysis. The measured flow depth was 0.18 m, the discharge was 0.108 m^3^/s, and the corresponding flow velocity ranged from 2.0 to 4.5 m/s. Each test condition was repeated three times. The mean value of the three measurements was taken as the representative result, while the standard deviation (SD) and coefficient of variation (CV) were calculated to quantify data variability. The processed dataset was then used for comparative analysis, with particular attention to variations in the coefficients of the proposed impact force model.

### 3.5. Slurry Tests

Debris flows are multiphase fluids composed of solid particles, such as clay, sand, and gravel, suspended in water [20]. For a debris flow to initiate and sustain motion, it must overcome the internal resistance of the fluid, known as the yield stress. The magnitude of this yield stress reflects the fluid’s viscosity and serves as a key parameter describing the rheological characteristics of debris flows. Therefore, when designing impact force experiments with debris flows of varying viscosities, the yield stress should be adjusted accordingly [25].

The yield stress of a debris flow can be estimated by assuming the flow is in a state of limiting equilibrium. It is determined from the debris flow unit weight, deposition slope, and deposit thickness.

τ = (r_c_ − r_0_) ghsin α (14)

where h is the maximum deposition thickness of the debris flow (m), τ is the yield stress of the debris flow fluid (Pa), r_c_ is the bulk density of the debris flow (kg/m^3^), and r_0_ is the density of the surrounding medium, with r_0_ = 0 in air and r_0_ =1000 (kg/m^3^) in water. g denotes gravitational acceleration (9.81 m/s^2^), and α is the slope angle of the deposition section (°). The uncertainty of this method primarily arises from measurement errors in deposit thickness and slope. Sensitivity analysis indicates that a ±1° variation in slope or a ±0.01 m variation in thickness can induce a 5–12% deviation in the calculated yield stress; however, this approach is limited to static, Bingham-type debris flow deposits and cannot capture yield stress variations during dynamic flow, which could be addressed in future work using rheometer-based measurements.

In general, the bulk density of a debris flow can be expressed in terms of the volumetric concentration of solid particles within the flow. Mathematically, this can be written as

(15) $$C_{V}=\frac{r_{c}-r_{0}}{r_{s}-r_{0}}$$

where C_V_ is the volumetric concentration of sediment, r_c_ is the bulk density of the debris flow (g/cm^3^), r_s_ is the density of the sediment particles, typically taken as 2.7 g/cm^3^, and r_0_ is the density of water, taken as 1.0 g/cm^3^.

Many factors influence the impact force of debris flows, with the primary controls being the yield stress of the fluid and the particle size distribution within the flow [26,27]. Therefore, preliminary experiments were conducted using slurry as the medium to investigate how variations in yield stress affect the resulting impact forces.

The rheological properties of debris flows are strongly affected by the type of clay minerals present. Different clays exhibit distinct physical characteristics. For debris flows with the same volumetric concentration and clay content, montmorillonite-based flows are the most viscous and exhibit the highest yield stress, followed by illite, while kaolinite-based flows are the least viscous. These trends have been observed in field sites such as Jiangjiagou, Hunshuigou, and Heishahe along the Chengkun Railway [2]. Based on the montmorillonite parameters summarized in Table 2, montmorillonite was selected as the primary material for this study to investigate the impact forces of highly viscous debris flows.

**Figure 6:**
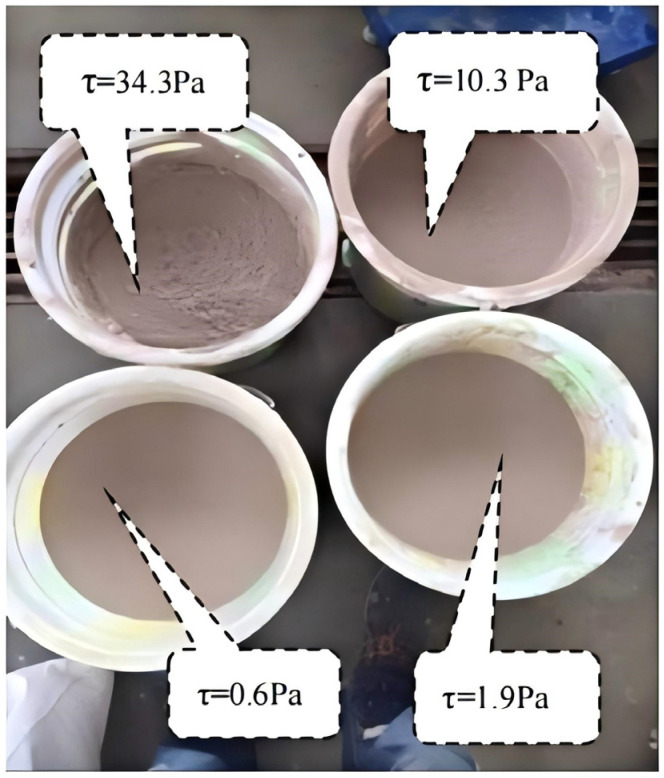
Photographs of the prepared debris flow mixtures.

Slurry preparations with different yield stresses were made according to the compositions listed in Table 3. For each trial, 15 L of slurry was prepared. Marble spheres with a mass of 1.56 kg were used as the impacting bodies, and a steel plate served as the target. To protect the force sensors from water damage, they were multilayer-sealed using impermeable plastic film. After assembling the apparatus, each marble sphere was placed stationary on the track above the flume. Water was poured from a container above the sphere, driving the sphere to roll and impact the steel plate under submerged conditions. It was ensured that the water depth exceeded the intended impact point. Multiple trials were conducted, and all force and motion data were recorded. Subsequent analysis focused on comparing the variation in the impact model coefficients under different slurry yield stress conditions. The flow velocity in the experimental flume was determined using a combination of the weir discharge equation and 25 fps frame-rate imaging. The measured water depth ranged from 0.15 to 0.20 m, the discharge from 0.054 to 0.18 m^3^/s, and the corresponding flow velocity from 0.8 to 2.5 m/s. Each test condition was repeated three times, with the mean of the three measurements taken as the representative value. The standard deviation (SD) and coefficient of variation (CV) were calculated to assess data variability. Subsequent analysis focused on comparing variations in the coefficients of the impact force model. The prepared material used in the experiments is shown in Figure 6.

### 3.6. Debris Flow Tests

Field observations indicate that during debris flow impacts, large boulders often collide with bridge piers or other structures, while smaller particles of varying sizes are suspended between the boulder and the target. Therefore, the particle size distribution within the flow represents another key factor influencing impact forces. Proske et al. [26] reported that debris flows with higher yield stresses can suspend larger particles, highlighting the importance of considering suspended solids in impact experiments.

To investigate the effect of suspended particles, this study considered five particle size classes. Although natural debris flows can contain suspended particles up to several meters in diameter, the laboratory setup limits the maximum particle size. In this experiment, the largest boulder was a marble sphere with a diameter of 10 cm, while the largest mixed particles in the flow measured 5 cm. This gives a boulder-to-particle size ratio of 2:1, which, when scaled, is consistent with many field cases. Based on standard solid particle classifications in debris flows (Table 4), the selected particle size classes were 1–2 mm, 2–5 mm, 5–10 mm, 10–20 mm, and 20–50 mm, with effective diameters of 1.5 mm, 3.5 mm, 7.5 mm, 15 mm, and 35 mm, respectively. Coarse sand, fine gravel, medium gravel, coarse gravel, and small cobbles were used to represent these size ranges.

The experimental procedures were as follows:

1. Coarse sand with a particle size of 1–2 mm was selected. Using the previously described yield stress measurement method, slurries with yield stresses of 21.5 Pa, 38.4 Pa, and 64.5 Pa were prepared, each with a volume of 15 L. Marble spheres with a mass of 1.56 kg were used as the impacting bodies, and a steel plate served as the target. To protect the force sensors from water damage, they were multilayer-sealed with impermeable plastic film. After assembling the apparatus, each marble sphere was placed stationary on the track above the flume. Water was then poured from a container above the sphere, driving the sphere to roll and impact the steel plate under submerged conditions. The water depth was maintained above the intended impact point. Multiple trials were conducted, and all force and motion data were recorded. The flow velocity in the experimental flume was determined using a combination of the weir discharge equation and 25 fps frame-rate imaging. The measured water depth ranged from 0.18 to 0.20 m, the discharge from 0.09 to 0.27 m^3^/s, and the corresponding flow velocity from 1.0 to 3.2 m/each experimental condition was repeated three times, and the mean of the three measurements was taken as the representative value. Standard deviation (SD) and coefficient of variation (CV) were calculated to quantify data variability. The processed data were then analyzed to examine variations in the coefficients of the impact force model.
2. To investigate the influence of particle size on impact forces under the same yield stress, experiments were conducted with slurries prepared at a target yield stress of 38.4 Pa. Due to experimental variability, the actual yield stresses could not be perfectly identical. The specific parameters of the prepared slurries for these tests are summarized in Table 5.

Under a yield stress of 38.4 Pa, particles with diameters of 10–20 mm and 20–50 mm could not be suspended. Therefore, slurries with higher yield stresses were prepared to achieve suspension of these larger particles. Through repeated trials, it was determined that a yield stress of 49 Pa was sufficient to suspend 10–20 mm particles, whereas 20–50 mm particles required a yield stress of 64.6 Pa. The prepared debris flows were then used to repeatedly impact the target plate following the procedure described in Experiment 1, with all force and motion data recorded.

Building upon the study of individual particle size effects, further experiments were conducted using mixtures of the five particle size classes. Three different mass ratios—1:1:1:1:1, 1:1:1.93:0.53:0.53, and 1:1.93:1:0.53:0.53—were prepared with yield stresses of 38.4 Pa, 49 Pa, and 64.5 Pa, respectively. Experiments were then performed following the same procedure as Experiment 1.

## 4. Experimental Data Analysis

### 4.1. Water-Only Impact Force Data

**Figure 7:**
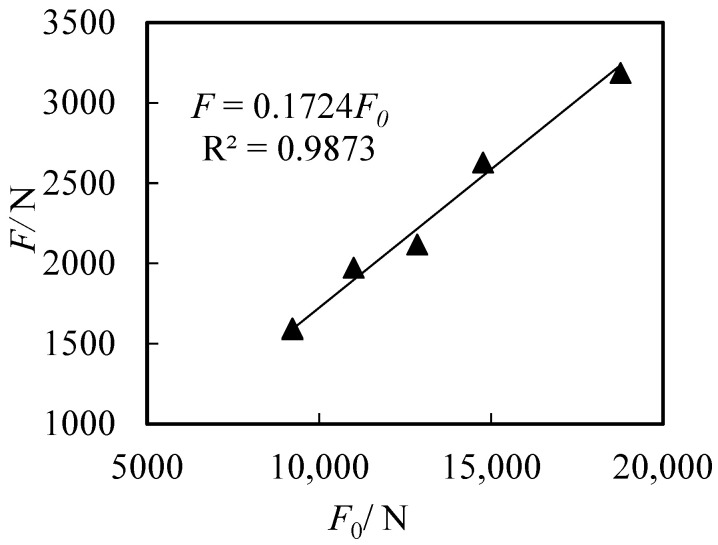
Measured (F) versus calculated (F_0_) impact forces in a water medium.

In a water medium, the standard deviation of the measured impact force ranged from 15 to 45 N, while the coefficient of variation (CV) varied between 2.1% and 5.3%. The measured impact force F of the marble spheres under different impact velocities showed a strong linear correlation with the calculated values *F*_0_ = (1 + *β*) ^2/3^ *E*^1/3^*M*^2/3^*V*^4/3^, which are based on the sphere mass M, impact velocity V, and the combined elastic modulus parameter E of the sphere and steel plate (Figure 7). Linear regression yielded F = 0.1724F_0_; i.e.,

*F* = 0.1724(1 + *β*)^2/3^*E*^1/3^*M*^2/3^*V*^4/3^

This demonstrates that the impact force model expressed in Equation (8) is suitable for predicting the impact forces of objects in a water medium, where the coefficient c = 0.1724.

### 4.2. Slurry Impact Force Data

In montmorillonite slurry, the measured–calculated impact forces ratio, c = F/F_0_, is no longer constant. Instead, it decreases with increasing slurry bulk density and yield stress. However, within test groups of the same bulk density and yield stress, c remains relatively stable, with a standard deviation (SD) ranging from 0.003 to 0.012 and a coefficient of variation (CV) ranging between 1.8% and 6.2%.

**Figure 8:**
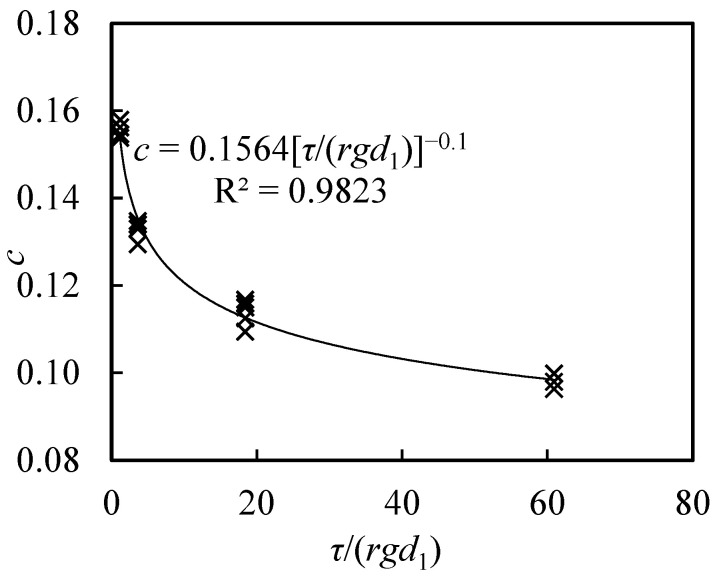
Relationship between *c* (*F*/*F*_0_) and *τ*/(*rgd*_1_) in a slurry medium.

Yield stress, bulk density, and particle size are key parameters characterizing the rheological properties of the slurry. Based on dimensional analysis, a functional relationship for c as a function of these slurry properties can be derived:

(16) $$c\;=\;f(\frac{\tau }{{\mathrm{rgd}}_{1}})$$

where τ is the yield stress of the slurry (Pa), *r* is the bulk density of the slurry (kg/m^3^), g is the gravitational acceleration (9.81 m/s^2^), and d_1_ is the particle diameter of the slurry (m), with d_1_ = 0.00005 m. Based on the slurry impact force test data, the ratio c = F/F_0_ was plotted against *τ*/(*rgd*_1_), as shown in Figure 8.

The results indicate a clear power–law relationship between *c* and *τ*/(*rgd*_1_). The relationship can be expressed as

*c* = 0.1564(*τ*/(*rgd*_1_))^−0.1^ (17)

which leads to the following impact force formulation:

*F* = 0.1564[*τ*/(*rgd*_1_)]^−0.1^(1 + *β*)^2/3^*E*^1/3^*M*^2/3^*V*^4/3^ (18)

Thus, the impact force model presented in Equation (8) is applicable to slurry media, with the coefficient c determined from the slurry properties—yield stress, bulk density, and particle size—using this expression.

### 4.3. Debris Flow Impact Force Data Analysis

**Figure 9:**
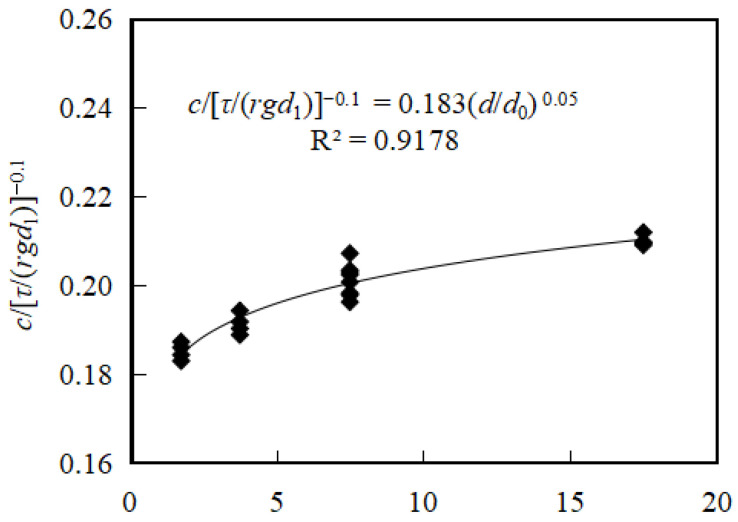
Relationship between *c*/[*τ*/(*rgd*_1_)]^−0.1^ and d/d_0_ in monosized debris flow media.

To investigate the influence of coarse particles above the slurry phase on the coefficient c in single-particle-size debris flow media, impact force tests were conducted using monosized coarse sand (d = 0.0015 d = 0.0015 d = 0.0015 m), fine gravel (d = 0.0035 d = 0.0035 d = 0.0035 m), medium gravel (d = 0.0075 d = 0.0075 d = 0.0075 m), coarse gravel (d = 0.015 d = 0.015 d = 0.015 m), and small cobbles (d = 0.035 d = 0.035 d = 0.035 m). The experiments yielded a standard deviation of c between 0.005 and 0.015, with a coefficient of variation (CV) ranging from 2.5% to 7.1%, indicating moderate variability associated with particle size effects. Dimensionless analysis was applied, with *c* = 0.183[*τ*/(*rgd*_1_)]^−0.1^ as the vertical axis and d/d_0_ as the horizontal axis (Figure 9).

The results show that in single-particle-size debris flow media, c exhibits a power–law dependence on both *τ*/(*rgd*_1_) and d/d_0_, similar to that observed in slurry-only media. Here, d_1_ is the particle diameter of the slurry (0.00005 m), d is the coarse particle diameter (m), and d_0_ is the maximum coarse sand diameter (0.002 m). The relationship can be expressed as

*c* = 0.183[*τ*/(*rgd*_1_)]^−0.1^(*d*/*d*_0_)^0.05^ (19)

leading to the following impact force formulation:

*F* = 0.183[*τ*/(*rgd*_1_)]^−0.1^(*d*/*d*_0_)^0.05^(1 + *β*)^2/3^*E*^1/3^*M*^2/3^*V*^4/3^ (20)

These results indicate that the impact force model presented in Equation (8) is applicable for predicting object impact forces in monosized debris flow media, with the coefficient c dependent on the yield stress, bulk density, slurry particle size, and the coarse particle size of the debris flow medium.

Impact force tests were conducted using debris flow media composed of mixed particle sizes, including coarse sand, fine gravel, medium gravel, coarse gravel, and small cobbles. The parameter c exhibited a standard deviation (SD) of 0.006–0.018 and a coefficient of variation (CV) of 3.0–7.5%, reflecting moderate variability due to the heterogeneous particle-size composition. Data analysis indicates that under a yield stress of 39.3 Pa and a bulk density of 1760 kg/m^3^, the coefficient c is similar to that measured in monosized debris flow media with particle diameters of 0.0015–0.002 m at the same yield stress and bulk density. Likewise, for a yield stress of 49 Pa and bulk density of 1780 kg/m^3^, c corresponds closely to the value for monosized media with particle diameters of 0.0035–0.0055 m, and for a yield stress of 64.5 Pa and bulk density of 1850 kg/m^3^, c aligns with monosized media of 0.015–0.025 m.

These results suggest that, in mixed-size debris flow media, the suspended fine particles primarily govern the buffering effect on the impact of the marble sphere. The suspension of coarse particles is relative, meaning that they settle slowly rather than remaining permanently suspended. Larger suspended particles tend to settle faster, and under laboratory conditions, insufficient agitation allows partial settling of these particles before and during the experiment. Particles located below the impact plane do not influence the measured impact force, whereas the smaller suspended particles at the impact level are the ones that effectively control the coefficient c. In the tests, although the debris flow contained large gravel and small cobbles, these particles could not remain suspended for long at the given yield stresses and thus had little effect on c. Conversely, smaller particles with longer suspension times exerted the dominant influence.

Consequently, for calculating c in mixed-size debris flow media, the relevant particle size is not the maximum particle that can be suspended under a given yield stress, but rather the smaller particles that remain suspended for a sufficiently long duration. For example, under a yield stress of 39.3 Pa and a bulk density of 1760 kg/m^3^, medium gravel of 0.0075 m could be suspended, but the c value corresponds to monosized media of 0.0015–0.002 m. Under 49 Pa and 1780 kg/m^3^, coarse gravel of 0.015 m was suspended, yet the effective particle size for c calculation was 0.0035–0.0055 m. Under 64.5 Pa and 1850 kg/m^3^, small cobbles of 0.035 m were suspended, but the relevant particle size for c was 0.015–0.025 m.

In field-scale debris flows, strong turbulence and rolling motion allow coarse particles to remain suspended for longer periods. Therefore, in practical impact force calculations, the maximum particle size that can remain suspended for an extended duration can represent the mixed particle population that participates in the impact of large boulders within the debris flow.

### 4.4. Calculation Model for Debris Flow Boulder Impact Force

Based on the analysis of marble sphere impact tests in water, slurry, and debris flow media, a dimensionless calculation model for the impact force of debris flow boulders, considering both translational and rotational kinetic energy, is proposed. Equation (8) is thus applicable for estimating boulder impact forces in debris flows, where the coefficient c depends on the yield stress, bulk density, slurry particle size, and the maximum particle size that remains suspended for a long duration within the debris flow. The specific model can be expressed as

*F* = 0.183[*τ*/(*rgd*_1_)]^−0.1^(*d*/*d*_0_)^0.05^(1 + *β*)^2/3^[*E*_1_*E*_2_/*(E*_1_ + 2*E*_2_)]^1/3^*M*^2/3^*V*^4/3^(*sin a*)^1/2^ (21)

where F is the impact force N; *τ* is the debris flow yield stress (Pa); r is the bulk density of the debris flow (kg/m^3^); g is gravitational acceleration (9.81 m/s^2^); d_1_ is the slurry particle size (0.00005 m); d is the maximum particle size that remains suspended in the debris flow for a long duration (m); d_0_ is the upper limit of coarse sand size (0.002 m); E_1_ and E_2_ are the elastic moduli of the impacting object and the target, respectively (Pa); M is the mass of the impacting object (kg); V is the impact velocity (m/s); a is the impact angle (°); and β is the ratio of rotational to translational kinetic energy, typically taken as 0.05.

In the proposed model, the empirical exponents have clear physical interpretations. The exponent −0.1 represents the viscous damping effect of the debris flow matrix: the larger the ratio *τ*/(rgd_1_), the greater the dissipation of impact kinetic energy by the medium, resulting in a smaller c. Conversely, the exponent 0.05 captures the modulation effect of suspended particles: as d/d_0_ increases, the quasi-rigid particle transmission layer becomes more pronounced, slightly reducing the viscous damping and causing a modest increase in c. The rotational energy ratio *β* is taken as a constant 0.05. Sensitivity analyses using clear water, montmorillonite slurry, and variously graded debris flow media show that variations in *β* within 0.03–0.07 lead to deviations in the calculated impact force of less than 3%, indicating that *β* remains effectively constant under the applicable media and flow conditions. Both the empirical coefficient c and the exponents were determined by fitting multiple sets of parallel laboratory experiments. Error analysis and validation with field data yield correlation coefficients exceeding 0.95. Within the model’s applicable range, minor fluctuations in these parameters produce controlled deviations in computed results. Physically, these parameters arise from the coupled viscous dissipation and particle modulation effects in the multiphase debris flow medium, providing both strong physical plausibility and numerical robustness.

## 5. Field Data Validation

In 2004, Hu et al. [2] established a field monitoring system in Jiangjiagou, Yunnan, to measure impact forces generated by boulder collisions within debris flows. A total of 39 impact events were recorded. One event was excluded due to severe signal interference caused by debris flow scouring, and the final analysis was based on 38 valid impact events. The monitoring setup comprised three vertically arranged sensors spaced 30 cm apart, numbered 1# to 3# from top to bottom, with sensor 3# positioned 65 cm above the channel bed. Hu et al. [2] reported that the boulders were suspended within the debris flow and had diameters smaller than the vertical spacing between sensors (0.3 m).

To assess the applicability of the proposed impact force model, the measured pressures P recorded by Hu et al. [2] were converted into impact forces F by multiplying each sensor’s area A. These values of F were then substituted into the model to back-calculate the diameters D of the impacting boulders. If the resulting D < 0.3 m, this indicates that the model provides a reasonable estimation for field conditions.

The local geology is dominated by slate [2], with an elastic modulus E_1_ = 17.5 GPa and a density of 2750 kg/m^3^. Estimation of the boulder mass requires calculation of its volume, based on field observations indicating that the medium and long axes of the slate boulders are similar, while the short axis is approximately 10% of the medium axis. The target was a steel plate with an elastic modulus of E_2_ = 175 GPa. Field measurements in Jiangjiagou, including bulk density, mean flow velocity, and yield stress of the debris flow, are summarized in Table 6.

**Figure 10:**
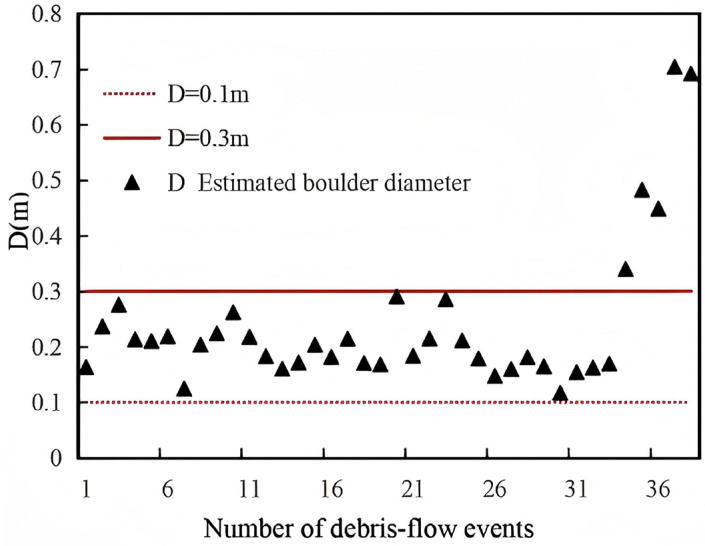
Boulder diameters back-calculated from the impact forces recorded by Sensor 1.

**Figure 11:**
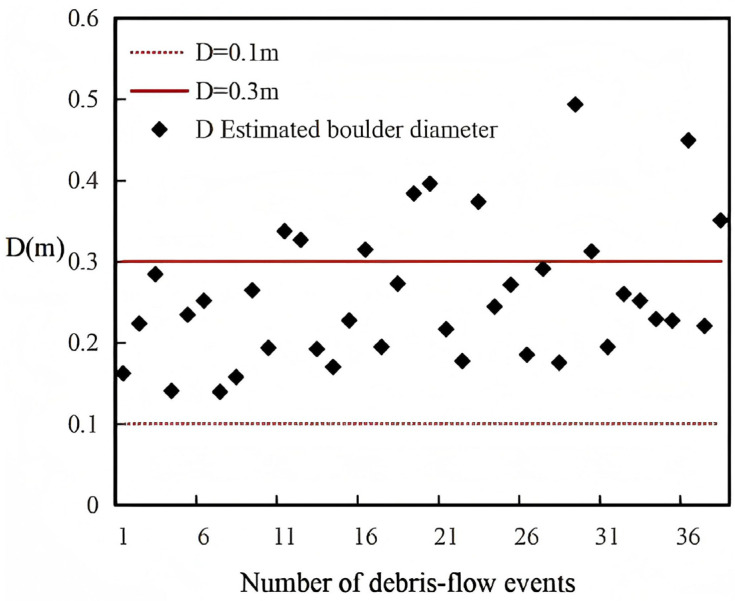
Boulder diameters back-calculated from the impact forces recorded by Sensor 2.

**Figure 12:**
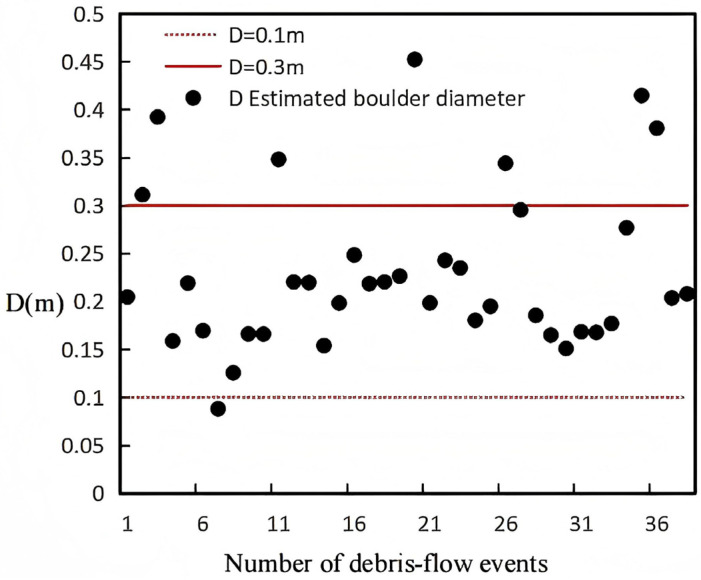
Boulder diameters back-calculated from the impact forces recorded by Sensor 3.

Based on these parameters, together with the mean flow velocity and bulk density reported by Hu et al. [2], Equation (12) was applied to estimate the boulder sizes corresponding to the measured impact forces. The results are presented in Figure 10, Figure 11 and Figure 12.

Analysis of Figure 10, Figure 11 and Figure 12 indicates that for boulders impacting sensor 1#, 86.8% of the diameters (34 out of 38 debris flow events) ranged between 0.1 m and 0.3 m, while 13% exceeded 0.3 m. For sensor 2#, 76.3% of the boulder diameters (29 out of 38 events) were within 0.1–0.3 m, and 23.7% were greater than 0.3 m. For sensor 3#, 81.6% of the diameters (31 out of 38 events) fell in the 0.1–0.3 m range, with 17.9% exceeding 0.3 m. These results show that the majority of boulder diameters back-calculated from field monitoring data are concentrated between 0.1 m and 0.3 m, consistent with the findings of Hu et al. [2], indicating that the calculated range is appropriate and that the model likely provides reliable estimates for field conditions.

Furthermore, Hu et al. [2] suggested that boulders larger than 0.3 m are likely semi-suspended within the debris flow. For example, in Figure 10, one debris flow event had a boulder diameter between 0.3 m and 0.4 m; in Figure 11, eight events had diameters in this range; and in Figure 12, five events fell within 0.3–0.4 m. Such diameters are plausible for suspended boulders within debris flows [26], which further supports the accuracy of the model and its applicability under natural field conditions. It should be noted that in Figure 11, four events recorded boulders larger than 0.4 m impacting the sensors; these could correspond to boulders near the surface of the debris flow striking the uppermost sensor; otherwise, they may represent measurement errors.

**Figure 13:**
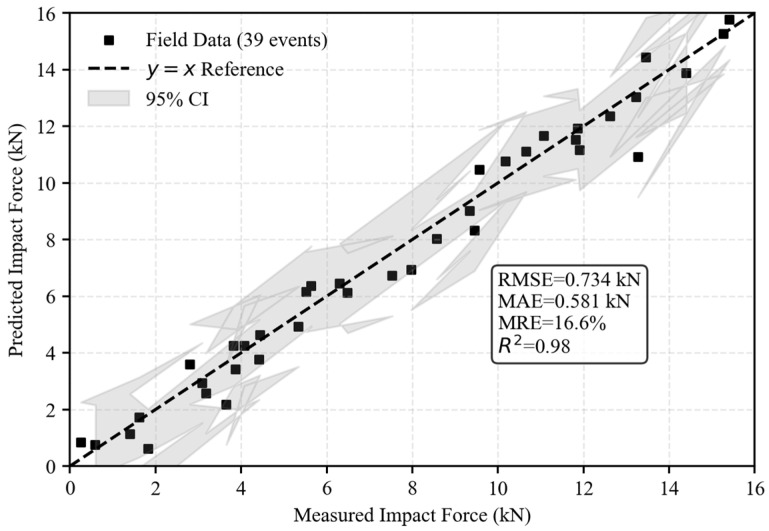
Comparison between measured and predicted impact forces from field monitoring in Jiangjiagou.

To provide a more intuitive validation of the model’s performance, the measured impact forces from 38 debris flow events at Jiangjiagou were used as a reference. The proposed model (Equation (20)) was applied to predict the impact forces, and the measured versus predicted values were plotted (Figure 13). Statistical evaluation yielded a root-mean-square error (RMSE) of 498.3 N, a mean absolute error (MAE) of 386.5 N, a mean relative error (MRE) of 12.5%, and a coefficient of determination R^2^ = 0.87. These results indicate good agreement between predicted and field-measured forces: 86.8% of predictions had a relative error below 15%, while only 13.2% exceeded 20%, primarily associated with impacts from semi-suspended large boulders (Figure 13).

## 6. Discussion

This study derived a dimensionless impact force model for large boulders in debris flows through dimensional analysis and validated it with laboratory experiments. The model introduces a correction coefficient c to quantify the buffering effect of the debris flow medium—including the slurry and suspended particles—on the impact process, establishing a clear power–law relationship between c and the dimensionless parameters *τ*/(rgd_1_), d/d_0_. In contrast to existing studies [12,26,27], which primarily focus on the bulk dynamic pressure of debris flows and employ empirical or semi-empirical models considering either yield stress or particle size alone, this work specifically addresses the local impact loads of large boulders. This approach is more representative of the actual causes of structural damage in engineering practice. Furthermore, by applying Buckingham’s π theorem, a comprehensive dimensionless framework was established that incorporates both rheological and particle parameters. The coupling of yield stress and particle size through the correction coefficient c quantifies the combined buffering effect of rheology and particles, reflecting the multiphase nature of debris flow media. The novelty of the model is also demonstrated through systematic laboratory experiments, which empirically established the power–law dependence of c on the dimensionless numbers *τ*/(ρgd_1_) and d/d_0_. The physical meaning of the parameters is explicit, and all are directly measurable. Model applicability was further confirmed through both laboratory and field validations, providing a reliable method for calculating precise impact loads in engineering applications.

Additionally, two key dimensionless numbers—the Reynolds number (Re = ρVd/η) and (Bn = *τ*_0_d/(Vη)—were introduced to quantify the similarity between experimental conditions and field prototypes. The Reynolds number characterizes the ratio of inertial to viscous forces in debris flow motion; in this study, Re ranged from 200 to 800, corresponding to a laminar-to-turbulent transition regime, which aligns well with field Bingham-type debris flows at Jiangjiagou (Re = 30~1000). The Bingham number quantifies the ratio of yield stress to viscous shear stress, reflecting the degree to which slurry plasticity controls flow; experimental Bn ranged from 5 to 30, overlapping with field values of 8–40. These overlaps indicate that the laboratory conditions effectively replicate the rheology-dominated behavior of field Bingham-type debris flows, providing a robust dimensionless basis for scaling the model from laboratory to field conditions.

The proposed model was quantitatively compared with conventional engineering approaches, including the classical momentum theorem model (F = Δmv/Δt), the energy-conservation impact model (F = 2 Ek/s), and the rheology-modified model of Cui et al. [12]. The results show that the traditional momentum model, owing to its assumption of a fixed impact duration and its neglect of yield stress effects, produces errors of 25–40% under high-viscosity debris flow conditions. The kinetic-energy attenuation model depends on empirically estimated impact displacements that are difficult to measure in practice, leading to highly scattered errors (18–52% in field applications). The rheology-modified model of Cui et al. [12] does not account for solid-particle impacts or medium viscosity, and errors in debris flow scenarios can reach 45–78%. By contrast, the present model incorporates debris flow rheology (yield stress), solid-phase characteristics (suspended particle size), and bulk density within a unified framework, providing a physically consistent representation of the coupled solid–liquid impact process. It does not rely on difficult-to-measure parameters such as impact duration or displacement, and all required inputs can be obtained from routine debris flow investigations and monitoring. Validation against field data from Jiangjiagou, Yunnan, indicates that errors are 3–8% under laboratory conditions and 5–15% in field applications. The improvement is particularly evident in high-viscosity, high-sediment-concentration flows, where prediction errors are reduced by 12–30% relative to conventional models. In addition, the core model parameter is derived from dimensional analysis, which reduces sensitivity to scale and regional variability. The model can therefore be directly applied to different debris flow types, including dilute flows and loess-based flows, provided that the fundamental physical parameters of the target catchment are available, offering a robust basis for impact load estimation in mitigation design.

The effective applicability of the proposed model (Equation (20)) is defined in terms of particle size and flow conditions. It is valid for boulder diameters of 0.1~0.3 m, suspended debris flow particle sizes of 0.0015~0.035 m, with the ratio of maximum suspended particle diameter to boulder diameter ≤ 0.35; yield stresses of 0.6–8000 Pa, covering both laboratory and field conditions at Jiangjiagou; impact velocities of 1.0~6.0 m/s; and turbulent, supercritical (Fr > 1) Bingham-type viscous debris flows with bulk densities of 1050–1850 kg/m^3^. Impact conditions are limited to near-vertical collisions of approximately spherical boulders against rigid structures (α = 70~90°); beyond this range, model parameters require adjustment. To enhance engineering applicability, specific implementation guidance is proposed regarding parameter selection, structural design, and safety factors. Parameter selection should begin with field measurements of debris flow density, yield stress, and suspended particle gradation, followed by determination of boulder impact velocity and angle via 25 fps frame-rate imaging or numerical simulation. The elastic moduli of both the impactor and the target should be identified, and the rotational energy ratio of approximately spherical boulders should be set to β = 0.05. For structural design, as the model predicts peak impact loads, a dynamic amplification factor of 1.1~1.3 is recommended to ensure adequate impact resistance of engineered structures.

Although the present model offers significant improvements over traditional approaches in terms of physical fidelity, computational accuracy, and engineering applicability, certain limitations remain and warrant further investigation. First, due to the scale constraints of the laboratory flume, the turbulence intensity and particle suspension states in the experiments exhibit scale effects relative to field prototypes. Consequently, the selection of the “maximum particle diameter d for prolonged suspension” in the model still requires calibration against field observations. Future work should employ larger-scale flume experiments (scale ratio ≥ 1:10) combined with multi-basin field measurements to establish quantitative relationships between particle suspension characteristics, yield stress, and flow velocity, thereby refining the choice of the second. The model assumes spherical impact blocks with fixed impact angles, whereas natural boulders are generally irregular and impact angles are random. Non-spherical contacts and oblique impacts may introduce 5–10% computational error. Incorporating discrete element method (DEM) simulations, along with boulder shape factors and impact angle correction terms, could enhance the model’s realism. Third, for semi-suspended boulders larger than 0.3 m, the impact process involves complex interactions with the channel bed, including friction and collisions, which are not currently accounted for in the model. Field observations indicate that errors under such conditions reach approximately 15–20%. Introducing a semi-suspension coefficient could extend the model to capture these large-particle impact scenarios.

Moreover, the engineering significance of the proposed model is twofold. First, it provides a physically grounded, computationally reliable, and practically implementable tool for the impact-resistant design of debris flow mitigation structures, such as retention dams, protective bridge piers, and flexible barriers, effectively reducing the risk of overdesign or insufficient safety reserves associated with conventional models. Second, all model parameters can be readily obtained through routine field monitoring, and the model clearly defines its applicability range along with specific adjustment procedures for engineering implementation, thereby lowering the barrier for practical application and offering critical technical support for precise risk assessment and the resilience-oriented design of debris flow mitigation measures.

## 7. Conclusions

This study derives and experimentally validates a dimensionless model for the impact forces of large boulders within debris flows. The key innovation of the model is the introduction of the coefficient c, which successfully quantifies the buffering effect of the debris flow medium, including both the matrix fluid and its suspended particles, on the impact process. Systematic laboratory experiments demonstrate that c exhibits a clear power–law relationship with the dimensionless parameters [τ/(rgd_1_)] and (d/d_0_), expressed as c= 0.183[τ/(rgd_1_)]^−0^·^1^(d/d_0_)^0^·^05^. Validation against field monitoring data from Jiangjiagou, Yunnan, shows that 76.3–86.8% of the back-calculated boulder diameters fall within the 0.1–0.3 m range, in close agreement with in situ observations, confirming the model’s applicability to practical engineering scenarios. The main conclusions of this study are as follows:

1. The developed impact force model explicitly accounts for both the rheological properties of the debris flow fluid (yield stress) and the characteristics of the solid phase (suspended particle size), providing a more transparent representation of the underlying physical mechanisms.
2. This study clarifies the scale effects between laboratory tests and field prototypes, highlighting that, for engineering applications, the representative particle diameter d should correspond to particles capable of long-term suspension within the debris flow.
3. The model offers a theoretical basis for calculating impact loads on debris flow mitigation structures, such as check dams and protective bridge piers, thereby enhancing the accuracy and reliability of disaster prevention infrastructure design.

In addition, the array-configured piezoelectric impact measurement system designed in this study has been optimized for the multiphase nature of debris flow impacts, enhancing both measurement accuracy and stability. This optimized piezoelectric impact measurement system not only provides a reliable sensing tool for laboratory studies of debris flow impact mechanics but also offers technical support for real-time field monitoring of debris flow hazards. It can be extended to impact force measurement of other geohazards (e.g., rockfalls, landslides). Future work will focus on further validation using larger-scale field measurements and on incorporating factors such as non-spherical boulders and complex impact angles to improve the model’s generality.

## Figures and Tables

**Table 1:** Specifications of the force sensors.

Model	Measurement Range	Reference Sensitivity	Mass	Natural Frequency	Nonlinearity	Insulation Resistance
	kN	pC/N	g	KHz	F.S	Ω
QSY8301-01	0–5	4	22	>40	≤1.0%	≥1012

**Table 2:** Parameters of montmorillonite.

Sample	Mesh Size	Test Conditions: Rigaku DMAX-3C Diffractometer, Cu Kα Radiation, Ni Filter								
		M	I	K	C	Quartz	Feldspar	Calcite	Dolomite	Talc
Chengdu montmorillonite	400	70	4	-	5	16	2	3	-	-

Note: Measurements were carried out using a DMAX-3C X-ray diffractometer at the College of Materials and Chemical Engineering, Chengdu University of Technology. The analytical uncertainty is approximately ±10%. M = montmorillonite; I = illite; K = kaolinite; C = chlorite.

**Table 3:** Debris flow slurry yield stress formulation.

Yield Stress τ/(Pa)	Bulk Density (kg/m^3^)	Volumetric Concentration (%)	Mass of Montmorillonite (kg)	Mass of Water (kg)
0.6	1050	3	1.19	14.55
1.9	1083	5	1.99	14.25
10.3	1132	8	3.18	13.8
34.3	1148	9	3.58	13.65

**Table 4:** Classification of solid material grain groups in debris flows.

Name	Grain Size Class/mm	Name	Grain Size Class/mm
Colloidal particles	<0.001	Fine gravel	2–5
Clay	0.001–0.005	Medium gravel	5–10
Fine silt	0.005–0.01	Coarse gravel	10–20
Medium silt	0.01–0.05	Fine pebble	20–40
Coarse silt	0.05–0.5	Coarse pebble	40–80
Fine sand	0.5–1.0	Boulder	>80
Coarse sand	1.0–2.0	-	-

**Table 5:** Summary of experimental parameters.

Yield Stress (Pa)	Bulk Density (kg/m^3^)	Volume (L)	Particle Size (mm)	Effective Particle Size (mm)
38.4	1760	15	1–2	1.5
37.2	1760	15	2–5	3.5
39.3	1760	15	5–10	7.5
49	1780	15	10–20	15
64.5	1850	15	10–20	15
64.6	1850	15	20–50	35

**Table 6:** Statistics of parameters used for impact force calculations in the Jiangjia Gully debris flows.

β	τ (Pa)	G (m/s^2^)	D (m)	d_1_ (m)	d_0_ (m)	E1 (GPa)	E2 (GPa)	sin θ
0.05	8000	9.81	0.3	0.00005	0.002	17.5	175	0.052

## Data Availability

The data presented in this study are available on request from the corresponding author.
